# The psychosocial assessment of heart transplant candidates in Ireland

**DOI:** 10.1192/j.eurpsy.2024.239

**Published:** 2024-08-27

**Authors:** K. Corrigan, G. Crudden, A. M. Clarke, A. Doherty, R. Duffy, Z. Azvee

**Affiliations:** ^1^St Vincent’s University Hospital; ^2^St. Vincent’s University Hospital; ^3^Holles Street, The National Maternity Hospital, Dublin; ^4^Naas General Hospital, Kildare; ^5^The Mater Misericordiae Hospital, Dublin, Ireland

## Abstract

**Introduction:**

We aim to describe the psychosocial features, including Stanford Integrated Psychosocial Assessment for transplantation (SIPAT) scores of individuals undergoing assessment for heart transplantation in Ireland.

**Objectives:**

All potential heart transplant candidates undergo assessment of psychosocial criteria to enhance selection and improve transplant outcomes. The Mater Hospital Consultation Liaison Psychiatry (CLP) department provides this national service in Ireland. All potential heart transplant candidates should receive a biopsychosocial assessment and screening via SIPAT tool as per international best practice. The SIPAT is a psychosocial evaluation and risk assessment tool which can help to determine suitability for organ transplant and identify modifiable risk factors to optimise a patient for transplant. Lower scores represent higher rates of suitability with a score < 21 representing an *acceptable* candidate and ≥21 *minimally acceptable.*

**Methods:**

We retrospectively examined the clinical files of all individuals referred to the national centre for heart transplant assessment over a five-year study period between January 2014 and December 2019.

**Results:**

One-hundred and fifty four individuals were referred for heart transplant assessment with 79% (n=122/154) listed for a heart transplant. The most common indication for heart transplant assessment was non-ischaemic cardiomyopathy (48%, n=74/154). Of those listed for transplant, 74% (n=90/122) went on to receive a heart transplant. Of those undergoing assessment for heart transplant, 92% (142/154) were assessed by CLP and 94% (144/154) received social work assessment.

SIPAT scores were available for 64/154 individuals with 22% (14/64) deemed *excellent* candidates for transplant, 59% (38/64) deemed *good* candidates, 14% (9/64) *minimally acceptable* candidate and 5% (3/64) deemed *high risk.* The SIPAT domain breakdown was as follows: patient readiness (mean 3.9, SD 3.4); social support system (mean 2.9, SD 4.2); psychological stability (mean 5.1, SD 4.9); and substance use (mean 3.8, SD2.4), with an average total score of 16 (SD 12.4).

Post-transplant, 26% (23/90) were referred and seen by CLP, 53% (48/90) were referred to social work and 32% (29/90) required psychology services. Seventeen individuals (19%, 17/90) received a psychiatric diagnosis and 27% (24/90) were prescribed psychotropic medication in the post-transplant period.

**Conclusions:**

This study describes for the first time the psychosocial factors and SIPAT scores of a national cohort of individuals referred for heart transplant. Psychiatric morbidity is high and this has implication for transplant suitability and post-operative course. This highlights the need for services to proactively identify and treat psychosocial factors in potential transplant recipients.

**Disclosure of Interest:**

None Declared

